# Diagnostic performance of gliomas grading and IDH status decoding A comparison between 3D amide proton transfer APT and four diffusion‐weighted MRI models

**DOI:** 10.1002/jmri.28211

**Published:** 2022-04-30

**Authors:** Hu Guo, Jun Liu, JunJiao Hu, HuiTing Zhang, Wei Zhao, Min Gao, Yi Zhang, Guang Yang, Yan Cui

**Affiliations:** ^1^ Department of Radiology The Second Xiangya Hospital, Central South University No. 139 Middle Renmin Road, Changsha Hunan 410011 China; ^2^ Department of Radiology Quality Control Center Hunan Province Changsha 410011 China; ^3^ MR Scientific Marketing, Siemens Healthineers Ltd. Wuhan 430071 China; ^4^ Department of Biomedical Engineering College of Biomedical Engineering & Instrument Science, Zhejiang University Hangzhou Zhejiang China; ^5^ Shanghai Key Laboratory of Magnetic Resonance School of Physics and Electronic, East China Normal University Shanghai China; ^6^ Department of Neurosurgery The Second Xiangya Hospital, Central South University No. 139 Middle Renmin Rd, Changsha Hunan Province 410011 P.R. China

**Keywords:** glioma grading, magnetic resonance imaging, IDH status, amide proton transfer imaging, advanced diffusion models

## Abstract

**Background:**

The focus of neuro‐oncology research has changed from histopathologic grading to molecular characteristics, and medical imaging routinely follows this change.

**Purpose:**

To compare the diagnostic performance of amide proton transfer (APT) and four diffusion models in gliomas grading and isocitrate dehydrogenase (IDH) genotype.

**Study Type:**

Prospective.

**Population:**

A total of 62 participants (37 males, 25 females; mean age, 52 ± 13 years) whose IDH genotypes were mutant in 6 of 14 grade II gliomas, 8 of 20 of grade III gliomas, and 4 of 28 grade IV gliomas.

**Field Strength/Sequence:**

APT imaging using sampling perfection with application optimized contrasts by using different flip angle evolutions (SPACE) and DWI with q‐space Cartesian grid sampling were acquired at 3 T.

**Assessment:**

The ability of diffusion kurtosis imaging, diffusion kurtosis imaging, neurite orientation dispersion and density imaging (NODDI), mean apparent propagator (MAP), and APT imaging for glioma grade and IDH status were assessed, with histopathological grade and genetic testing used as a reference standard. Regions of interest (ROIs) were drawn by two neuroradiologists after consensus.

**Statistical Tests:**

T‐test and Mann–Whitney U test; one‐way analysis of variance (ANOVA); receiver operating curve (ROC) and area under the curve (AUC); DeLong test. *P* value < 0.05 was considered statistically significant.

**Results:**

Compared with IDH‐mutant gliomas, IDH‐wildtype gliomas showed a significantly higher mean, 5th‐percentile (APT_5_), and 95th‐percentile from APTw, the 95th‐percentile value of axial, mean, and radial diffusivity from DKI, and 95th‐percentile value of isotropic volume fraction from NODDI, and no significantly different parameters from DTI and MAP (*P* = 0.075–0.998). The combined APT model showed a significantly wider area under the curve (AUC 0.870) for IDH status, when compared with DKI and NODDI*.* APT_5_ was significantly different between two of the three groups (glioma II vs. glioma III vs. glioma IV: 1.35 ± 0.75 vs. 2.09 ± 0.93 vs. 2.71 ± 0.81).

**Data conclusion:**

APT has higher diagnostic accuracy than DTI, DKI, MAP, and NODDI in glioma IDH genotype. APT_5_ can effectively identify both tumor grading and IDH genotyping, making it a promising biomarker for glioma classification.

**Evidence Level:**

1

**Technical Efficacy:**

Stage 2

Gliomas account for approximately 77% of primary malignant brain tumors.^1^ The classification of gliomas has been mainly based on histogenesis.^2^ According to the 2016 WHO criteria, molecular features should be incorporated into the classification of brain tumors.^3^ For diffuse astrocytic and oligodendroglia tumors, isocitrate dehydrogenase (IDH) genotypes are important for subtyping.^4^ Clinical management and prognosis differ greatly in gliomas with different grades, pathological types, and genotypes.^5^, ^6^

MRI is the reference standard for imaging characterization of gliomas, and it is frequently used in the diagnosis and posttreatment management of patients with gliomas.^7^ Accurate grading and IDH genotype from neuroradiological assessments of gliomas based on MRI can help primary diagnosis and posttherapeutic follow‐up.^8^

However, MRI images from conventional sequences, may show similar morphologic findings among different types of gliomas, are less accurate than the results from histopathology in the detection of glioma grading and IDH genotype. Molecular imaging may provide additional complementary information to assist in diagnosis, which may help improve patient outcomes.^8^

Amide proton transfer imaging (APT) MRI has previously been used to assist in tumor grading and IDH‐mutant status by reflecting biologically active tumor portion with high cellularity and proliferation.^9^ Furthermore, a novel whole‐brain isotropic‐resolution chemical exchange saturation transfer (CEST) sequence using an optimized three‐dimensional turbo spin echo (TSE) readout sequence with negligible susceptibility artifacts has been shown to improve acquisition efficiency and image quality, which may increase the feasibility and reliability of clinical CEST.^10^

Diffusion‐weighted imaging (DWI) is a valuable imaging biomarker for classifying gliomas, as it allows for an assessment of the tumor microenvironment.^11^ Recently, MRI diffusion models, such as diffusion kurtosis imaging (DKI), neurite orientation dispersion and density imaging (NODDI), and the non‐Gaussian‐based mean apparent propagator (MAP)‐MRI, have been used to assess gliomas tissue.^12^, ^13^, ^14^, ^15^

APT and DWI imaging are particularly effective in grading glioma and detecting IDH‐mutant status in patients because they represent the changes of the tissue molecular levels, do not require contrast agents, and provide different diagnostic information from routine clinical sequences.^16^, ^17^ Therefore, this study aimed to compare the diagnostic efficacy of the five models (DTI, DKI, NODDI, MAP, and APT imaging) in evaluating tumor grades and genotype IDH status and to locate the best imaging indicators for aiding accurate diagnoses and treatment decisions.

## Materials and Methods

### Patient Recruitment

This prospective study was approved by the hospital ethics committee. All patients signed an informed consent form. Patients with histopathologically proven gliomas, World Health Organization grade II, III, or IV, were enrolled from May 2020 to August 2021. Inclusion criteria for patients with gliomas were as follows: 1) MRI scans had been performed before the patients were initially treated; 2) patients underwent complete multiparametric MRI examinations consisting of T2 fluid attenuated inversion (FLAIR), DWI, APT, and precontrast and postcontrast 3D T1‐weighted (T1w) magnetization prepared rapid gradient echo (MPRAGE) imaging; and 3) The IDH‐mutant status of the genotypes of the patients was acquired from the surgical resections after MRI examination. Finally, a total of 62 patients were included.

### Image Acquisition

MRI examinations were performed on a 3 T MRI scanner (MAGNETOM Skyra; Siemens Healthcare, Erlangen, Germany) with a 32‐channel head coil. Imaging sequences were as follows: 1) axial precontrast T2 FLAIR‐weighted TSE sequence (repetition time [TR] = 9000 msec, echo time [TE] = 99 msec, matrix size = 320 × 224, field of view [FOV] = 230 mm × 200 mm, slice thickness = 5 mm); 2) DWI; 3) sagittal APT imaging; and 4) axial precontrast and postcontrast T1w MPRAGE sequence (TR = 2000 msec, TE = 2.45 msec, matrix size = 256 × 256, FOV = 256 mm × 256 mm, slice thickness = 1 mm). The APT imaging was based on a whole‐brain isotropic‐resolution CEST sequence, known as the “sampling perfection with application optimized contrasts by using different flip angle evolutions” (SPACE).^10^ It used the following scan parameters: FOV = 212 × 212 × 201 mm^3^, matrix = 76 × 76 × 72, resolution = 2.8 × 2.8 × 2.8 mm^3^, TR = 3 seconds, TE = 17 msec, turbo factor = 140, number of averages (NSA) = 1.2, and generalized autocalibrating partially parallel acquisition (GRAPPA) factor = 2 × 2. For the APT imaging, B_0_ shimming sequence with a duration of 54 seconds was used to improve the field homogeneity in the brain region. The second‐order advanced shimming was utilized with the shimming target set only in the brain region. Then, the CEST saturation module consisted of ten 100‐msec‐long Gaussian pulses, each with a root mean square power of 2.5 μT. Seven CEST saturation offsets for APTw imaging were executed, including unsaturated (S_0_) and saturated frequencies of ±3 ppm, ±3.5 ppm, and ± 4 ppm, with the total acquisition duration at 4 minutes 38 seconds. A diffusion‐weighted MRI was performed in the transverse plane using a half q‐space Cartesian grid diffusion model with a radial grid size of 3 (62 diffusion directions and 10 different b‐values). The following parameters were used: TR = 4500 msec, TE = 111 msec, 60 slices, FOV = 192 × 192 mm^2^, matrix = 96 × 96, resolution = 2.0× 2.0 × 2.0 mm^3^, and b = 0, 350, 650, 1000, 1350, 1650, 2000, 2650, 2700, 3000 sec/mm^2^. The acquisition time of the DWI was 5 minutes 12 seconds.

## Data Analysis

### APT Processing

APT source images were registered to the 3D T1w MPRAGE image using the Statistical Parametric Mapping (SPM12) toolbox (http://www.fil.ion.ucl.ac.uk/spm) in MATLAB (R2017a, MathWorks, Inc., Natick, MA, USA), with a rigid body transformation. The APT and B_0_ source images were then spatially interpolated to a nominal resolution of 1.4 × 1.4 × 1.4 mm^3^. The magnetization transfer ratio was computed on the MATLAB platform by calculating MTR_asym_ at 3.5 ppm on a voxel‐by‐voxel basis as follows:

$${\mathrm{MTR}}_{\text{asym}}\;\left(3.5\;\mathrm{ppm}\right)=S_{\mathrm{sat}}\;\left(-3.5\;\mathrm{ppm}\right)/S_{0}-S_{\mathrm{sat}}\;\left(3.5\;\mathrm{ppm}\right)/S_{0}.$$

where S_sat_ and S_0_ are the image signal intensities measured with and without radiofrequency saturation pulse. The APTw images were generated by subtracting the corrected images.^10^

### Diffusion Processing

Diffusion‐weighted data initially underwent eddy current and motion corrections using the Diffusion Kit eddy tool.^18^ Then, the parameters of the four diffusion models were calculated using the in‐house NeuDiLab software based on the open‐resource tool DIPY (http://dipy.org). The calculation of the DTI model used b = 0, 650, 1000 data and the rest three models used all the b value data. The parameters derived included the axial, radial, and mean diffusivity (AD, RD, MD) and fractional anisotropy (FA) from DTI and DKI; the axial, radial, and mean kurtosis (AK, RK, MK) from DKI; the mean squared displacement (MSD), q‐space inverse variance (QIV), axial, radial and mean non‐Gaussianity (NGax, NGrad, NG), the return‐to‐plane probability (RTPP), the return‐to‐axis probability (RTAP), and the return‐to‐origin probability (RTOP) from MAP‐MRI; the intracellular volume fraction (ICVF), the isotropic volume fraction (ISOVF) and the orientation dispersion index (ODI) from NODDI. After diffusion calculation, the b = 0 images were registered with T1w MPRAGE to generate the transform matrix. Then, all diffusion parameter maps are registered to the T1w MPRAGE image through the transformation matrix.

### Image Analysis

All the images were resampled to the exact resolution of 1 × 1 × 1 mm^3^ and registered to the precontrast T1w MPRAGE images by linear interpolation using the “flirt” tool in FSL (V6.0; FMRIB Oxford University). After registration, a subtraction was performed between precontrast and postcontrast T1w MPRAGE images using the “fslmaths” tool in FSL (V6.0; FMRIB Oxford University). Two neuroradiologists (J.L and W.Z, who had 15 and 6 years of experience in neuroradiology, respectively) carefully delineated regions of interest (ROI) after consensus using ITK‐SNAP v.3.8.0.^19^ Postcontrast T1w MPRAGE, subtraction T1w MPRAGE and T2w FLAIR images were used as references to ensure that ROI included active tumor tissue but excluded cystic, necrotic, or hemorrhagic areas.^20^ For each patient, three to five ROIs (according to the tumor sizes, approximately 40 mm^2^ each on one image or on different planes) were drawn. The DWI and the APT imaging results were calculated for the entire ROI using the “fslstats” tool in FSL (V6.0; FMRIB Oxford University), which were transferred from the reference maps of T2w FLAIR and post‐contrast T1w images. A histogram approach in the segmented region was used to obtain the mean, the 5th‐percentile and 95th‐percentile of values. The histogram approach was based on the concept that the 5th and 95th percentiles of values, which are less affected by random statistical fluctuations, can be analogous to the minimum and maximum values.^8^, ^21^, ^22^

### Statistical Analysis

The metric variables are expressed as means ± standard deviation. All statistical analyses were carried out using SPSS (v. 23.0; Chicago, IL, USA). For the comparison between IDH‐mutant and IDH‐wildtype groups, the distribution of each MR variable was first assessed using the Shapiro–Wilk test. Then the two‐tailed independent samples t‐test was performed for normal distribution data and Mann–Whitney U test for non‐normal distribution data. The statistical significance was defined as a *P* < 0.05. One‐way ANOVA was utilized to assess the differences among the three glioma grades. For the significantly different parameters, the homogeneity of variance test was performed; and then the post hoc least significant difference (LSD) methods for equal variance data and Dunnett's T3 for unequal variance data. The parameters from the APT, DTI, DKI, NODDI, and MAP with *P* < 0.05 in identification of IDH genotypes and grading were selected to set up the corresponding combined models using the logistic regression method. Receiver operating characteristic (ROC) curves were drawn, with sensitivity, specificity, area under the curve (AUC), and the corresponding optimal thresholds used to assess the diagnostic performance of each significantly different parameters and the combined models. The DeLong test was performed to compare the AUCs of the models.

## Results

### Study Population

Patient demographics are summarized in Table 1. Sixty‐two patients (25 females and 37 males, mean age, 52 ± 13 years, ranges 19–78 years), who met the eligibility criteria by their medical records, were enrolled. Forty‐eight patients (32 males and 16 females; age, 19–78 years) with high‐grade gliomas, including 20 patients with WHO grade III and 28 patients with WHO grade IV, and 14 patients (5 males and 9 females; age, 30–72 years) with low‐grade gliomas were diagnosed by histopathology. IDH‐wildtype was found in 44 patients (71%), of which 8 were categorized as WHO grade II, 12 as WHO grade III, and 24 as WHO grade IV.

**TABLE 1:** Distribution of Demographic and Related Tumor Characteristics of Cases

	All Patients	Grade II	Grade III	Grade IV
No. of patients	62	14	20	28
Age (years)				
Mean	51.9	51.5	51.8	52.1
Range	19–78	30–72	19–75	19–78
Sex				
Male	37	5	12	20
Female	25	9	8	8
IDH status				
Wildtype	44	8	12	24
mutant	18	6	8	4

IDH = isocitrate dehydrogenase.

### Diagnostic Performances About IDH Status Decoding of Diffusion, and Amide Proton Transfer Metrics

**FIGURE 1:**
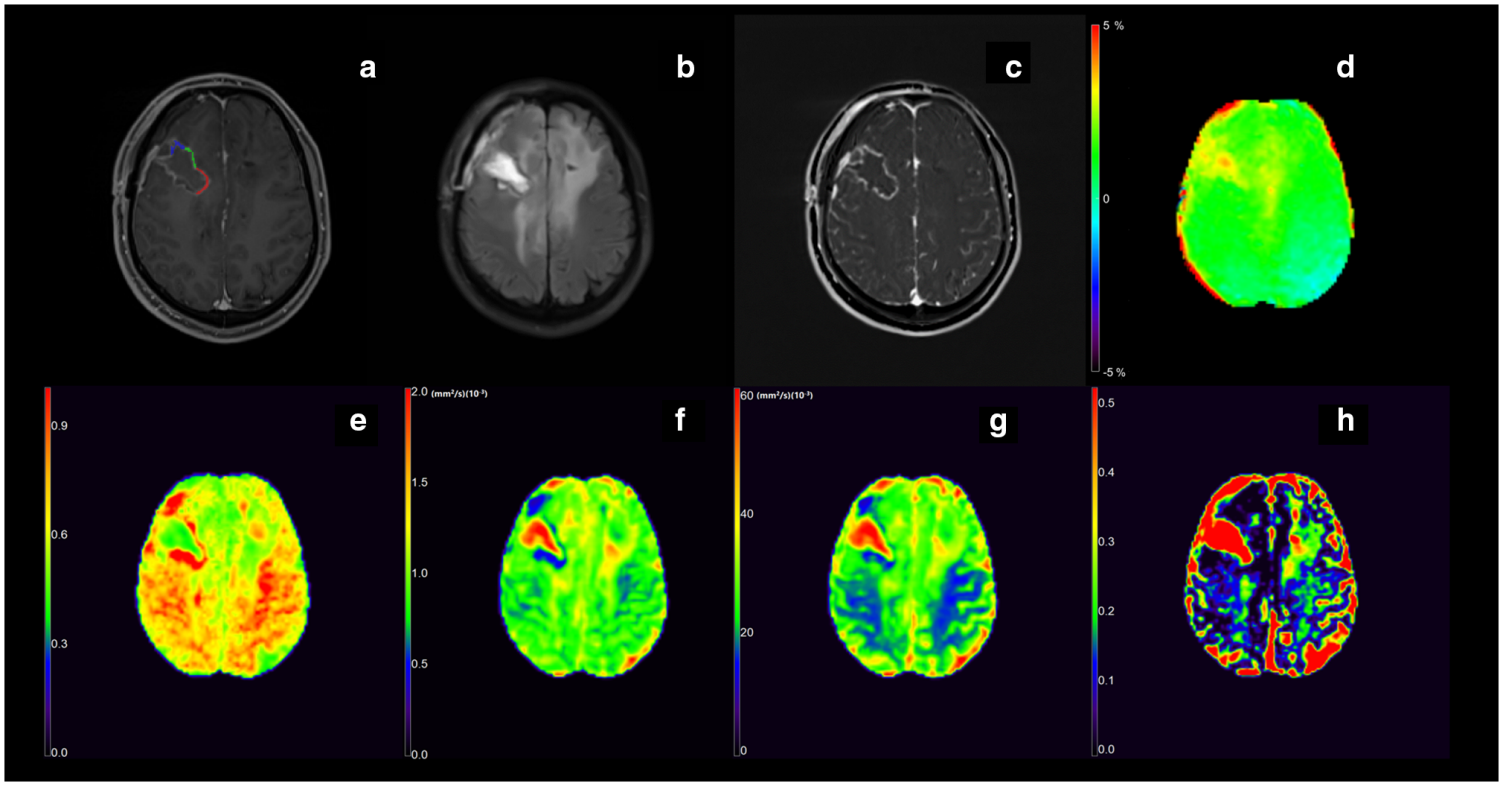
Images from a 49‐year‐old man with histologically proven oligodendroglioma IDH‐mutant (WHO grade III), including T1W image (a), T2 FLAIR image (b), Subtract image between T1W postcontrast and precontrast (c), APTw (d), DKI_AK (e), DTI_AD (f), MAP_MSD (g), NODDI_ISOVF (h). Positions of the regions of interest (blue, green, and red overlays) used in this participant are indicated in T1W image (a). The unit of DTI_AD and MAP_MSD is (mm^2^/sec) (×10^−3^).

**FIGURE 2:**
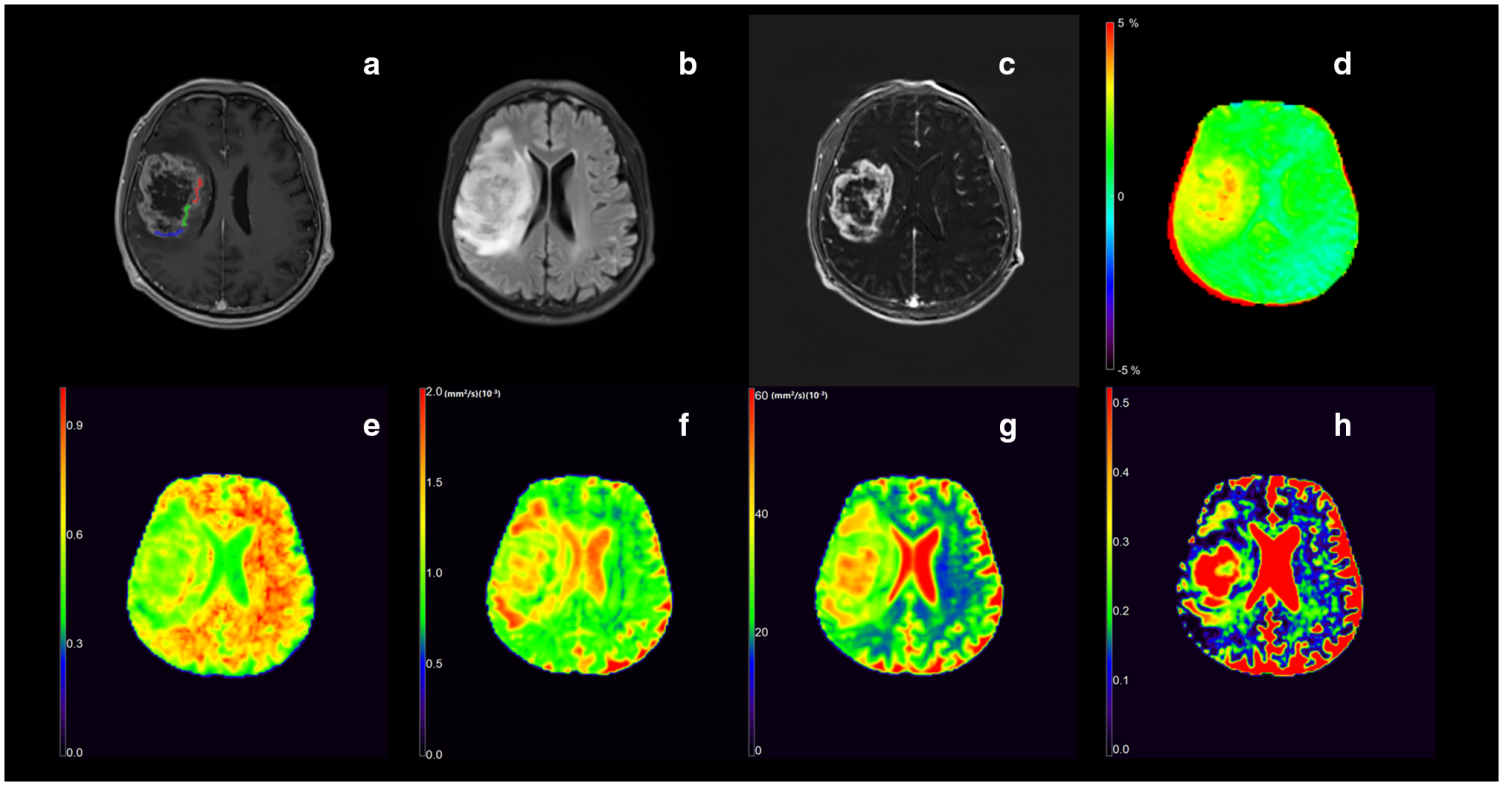
Images from a 63‐year‐old man with histologically proven astrocytoma IDH‐wildtype (WHO grade III), including T1W image (a), T2 FLAIR image (b), subtract image between T1W postcontrast and precontrast (c), APTw (d), DKI_AK (e), DTI_AD (f), MAP_MSD (g), NODDI_ISOVF (h). Positions of the regions of interest (blue, green, and red overlays) used in this participant are indicated in T1W image (a). The unit of DTI_AD and MAP_MSD is (mm^2^/sec) (×10^−3^).

**FIGURE 3:**
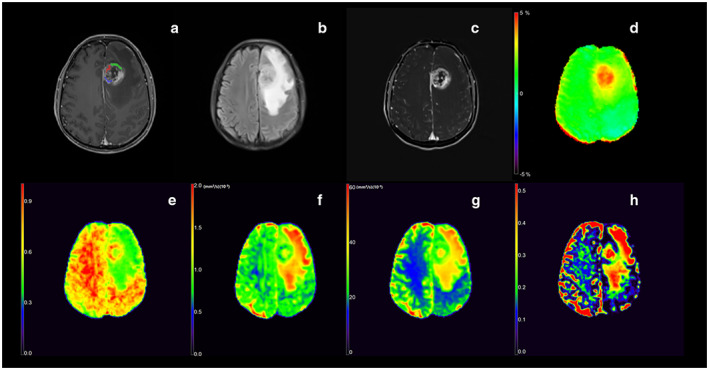
Images from a 52‐year‐old man with histologically proven glioblastoma IDH‐wildtype (WHO grade IV), including T1W image (a), T2 FLAIR image (b), Subtract image between T1W postcontrast and precontrast (c), APTw (d), DKI_AK (e), DTI_AD (f), MAP_MSD (g), NODDI_ISOVF (h). Positions of the regions of interest (blue, green, and red overlays) used in this participant are indicated in T1W image (a). The unit of DTI_AD and MAP_MSD is (mm^2^/sec) (×10^−3^).

**FIGURE 4:**
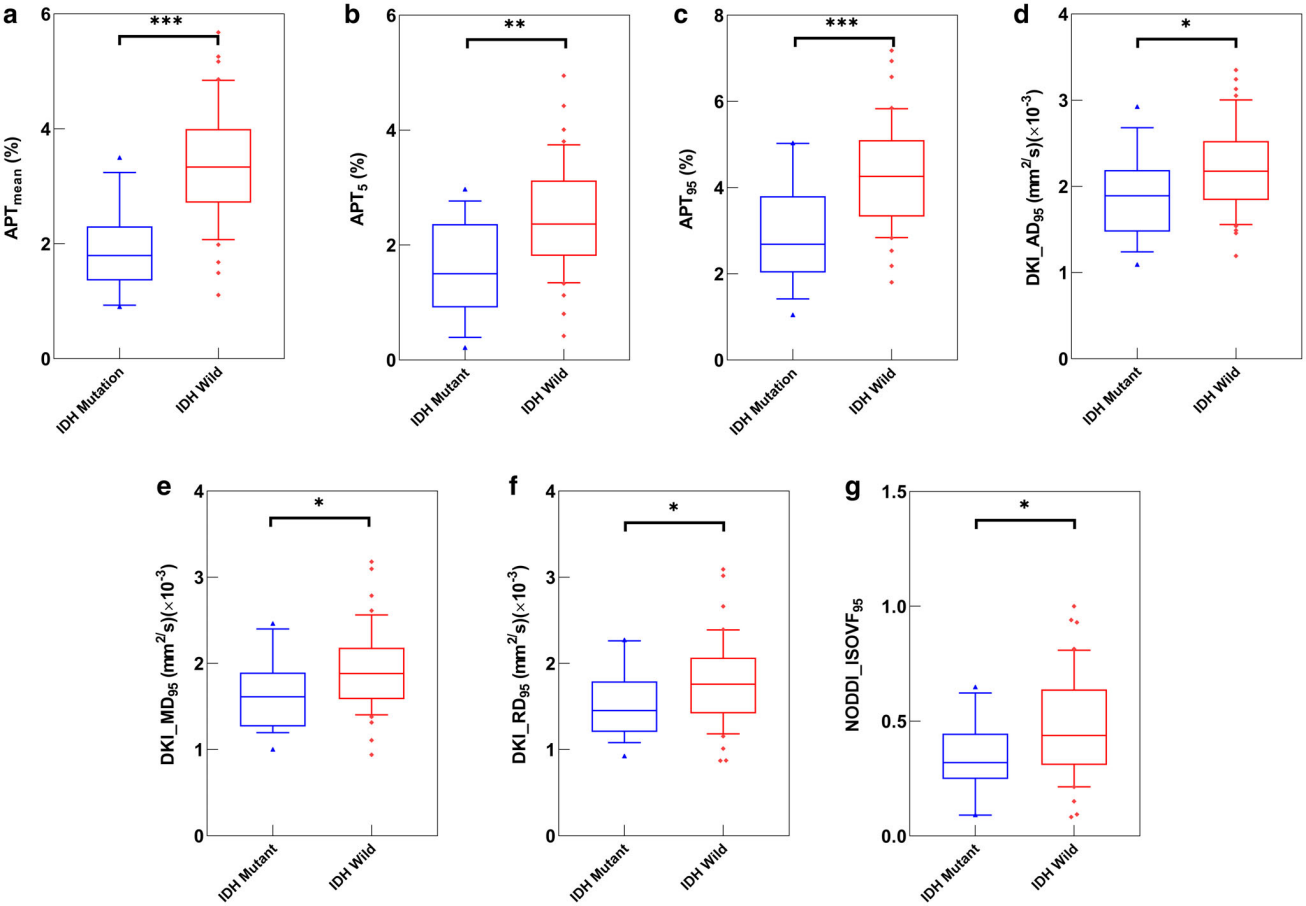
Box plots of APT_mean_ (a), APT_5_ (b), APT_95_ (c), DKI_AD_95_ (d), DKI_MD_95_ (e), DKI_RD_95_ (f), and NODDI_ISOVF_95_ (g) metrics in gliomas stratified according to isocitrate dehydrogenase (IDH) status (IDH‐mutant and IDH‐wildtype). Horizontal line indicates median, and bottom and top edges of box indicate 10th and 90th percentiles, respectively. Outliers are plotted individually by using triangle. IDH = isocitrate dehydrogenase. APT = amide proton transfer. DKI = diffusion kurtosis imaging. NODDI = neurite orientation dispersion and density imaging. AD = axial diffusivity. MD = mean diffusivity. RD = radial diffusivity. ISOVF = isotropic volume fraction. *5 = 5‐percentile value of * signal. *95 = 95‐percentile value of * signal. * = statistically significant difference (*P* < 0.05). ** = statistically significant difference (*P* < 0.01)

Images from conventional MRI, APT MRI, and the four diffusion models of representative cases of IDH‐mutant WHO grade III, IDH‐wildtype WHO grade III, and IDH‐wildtype WHO grade IV are shown in Figs. 1, 2, 3. Compared to IDH‐mutant gliomas, IDH‐wildtype gliomas showed significantly higher mean (3.38 ± 1.02 vs. 1.93 ± 0.77), 5th‐percentile (2.45 ± 0.94 vs. 1.60 ± 0.84), and 95th‐percentile values (4.3 ± 1.19 vs. 2.91 ± 1.22) of APT (APT_mean_, APT_5_, APT_95_); AD_95_ ([2.20 ± 0.50] × 10^−3^ mm^2^/sec vs. [1.88 ± 0.49] × 10^−3^ mm^2^/sec), MD_95_ ([1.93 ± 0.48] × 10^−3^ mm^2^/sec vs. [1.64 ± 0.41] × 10^−3^ mm^2^/sec), and RD_95_ ([1.80 ± 0.50] × 10^−3^ mm^2^/sec vs. [1.52 ± 0.39] × 10^−3^ mm^2^/sec) from DKI; and ISOVF_95_ (0.29 ± 0.18 vs. 0.20 ± 0.12) from NODDI. These results are shown in Table 2 and Fig. 4. There were no significantly different parameters from DTI and MAP diffusion models (*P* = 0.075–0.998), as shown in Appendix 1 of the Supplementary Material.

**TABLE 2:** The Parameters Derived From APT, DKI, and NODDI (mean ± standard deviation) in Different IDH Status

Parameters	IDH‐wildtype (*n* = 44)	IDH‐mutant (*n* = 18)	*P* value
APT_mean_	3.38 ± 1.02	1.93 ± 0.77	<0.0001***
APT_5_	2.45 ± 0.94	1.60 ± 0.84	0.002*
APT_95_	4.30 ± 1.19	2.91 ± 1.22	<0.001**
DKI_AD_95_ (10^−3^ mm^2^/sec)	2.20 ± 0.50	1.88 ± 0.49	0.023*
DKI_MD_95_ (10^−3^ mm^2^/sec)	1.93 ± 0.48	1.64 ± 0.41	0.031*
DKI_RD_95_ (10^−3^ mm^2^/sec)	1.80 ± 0.50	1.52 ± 0.39	0.039*
NODDI_ISOVF_95_	0.29 ± 0.18	0.20 ± 0.12	0.017*

IDH = isocitrate dehydrogenase. APT = amide proton transfer. DKI = diffusion kurtosis imaging. NODDI = neurite orientation dispersion and density imaging. AD = axial diffusivity. MD = mean diffusivity. RD = radial diffusivity. ISOVF = isotropic volume fraction. *5 = 5th‐percentile value of * signal. *95 = 95th‐percentile value of * signal.

* *P* < 0.05.

** *P* < 0.001.

*** *P* < 0.0001.

**FIGURE 5:**
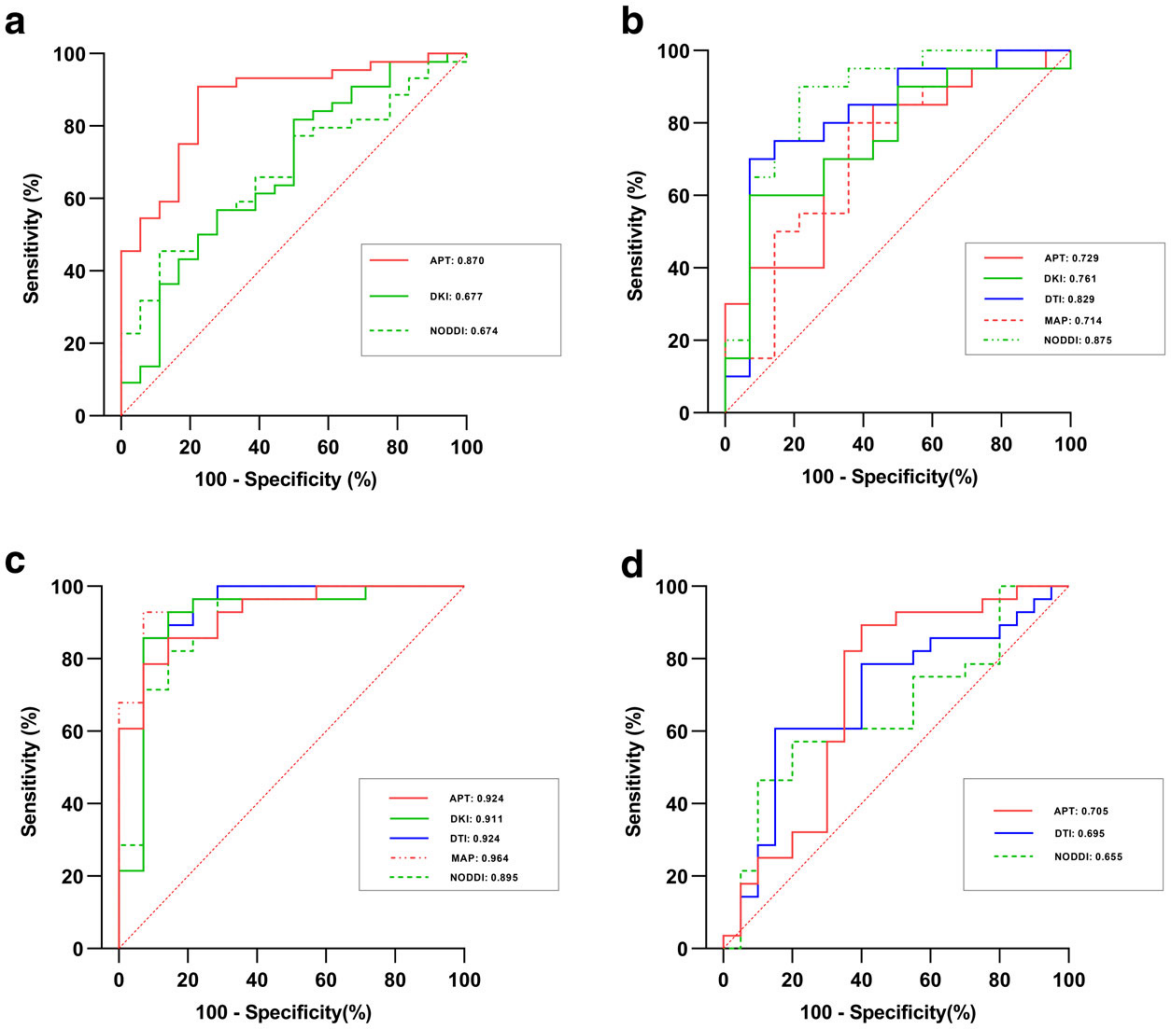
Receiver operating characteristic (ROC) analyses of APT, DKI, DTI, MAP and NODDI for comparisons: (a) IDH‐mutant vs. IDH‐wildtype, (b) grade II vs. grade III, (c) grade II vs. grade IV, (d) grade III vs. grade IV. APT = amide proton transfer. DKI = diffusion kurtosis imaging. DTI = diffusion tensor imaging. MAP = mean apparent propagator. NODDI = neurite orientation dispersion and density imaging.

The above seven significantly different metrics had AUCs ranging from 0.674 (NODDI_ISOVF_95_) to 0.870 (APT_mean_), and the associated AUC, accuracy, sensitivity, specificity, positive predictive, negative predictive, and best cutoff values for each parameter and their correspondingly combined models are shown in Table 3. Receiver operating characteristic curves of the combined models are shown in Fig. 5a. Through the Delong test of pairwise comparison for the above seven parameters, only APT_mean_ had significantly higher diagnostic performances than the APT_5_, DKI_MD_95_, DKI_RD_95_, and NODDI_ISOVF_95_, as reported in Appendix 2 of the Supplementary Material. In addition, the combined APT model had a significantly larger AUC (0.870) than DKI (0.677) and NODDI (0.674) models, as shown in Appendix 2 of the Supplementary Material and Table 5.

**TABLE 3:** Performance of All Considered Metrics in the Comparison of IDH Mutant and IDH Wildtype

Parameters	APT_all_	APT_mean_	APT_5_	APT_95_	DKI_all_	DKI_AD_95_	DKI_MD_95_	DKI_RD_95_	NODDI_ISOVF_95_
AUC	0.870	0.867	0.736	0.793	0.677	0.686	0.677	0.674	0.674
Accuracy (%)	87.10	87.10	74.19	80.65	72.58	74.19	58.06	75.81	58.06
Sensitivity (%)	77.78	77.78	61.11	66.67	50.00	50.00	83.33	50.00	88.89
Specificity (%)	90.91	90.91	79.55	86.36	81.82	84.09	47.73	86.36	45.45
PPV (%)	77.78	77.78	55.00	66.67	52.94	56.25	39.47	60.00	40.00
NPV (%)	90.91	90.91	83.33	86.36	80.00	80.43	87.50	80.85	90.91
Best Cut‐off value	‐	2.14	1.76	3.11	‐	1.75	1.93	1.36	0.50

IDH = isocitrate dehydrogenase. APT = amide proton transfer. DKI = diffusion kurtosis imaging. NODDI = neurite orientation dispersion and density imaging. AD = axial diffusivity. MD = mean diffusivity. RD = radial diffusivity. ISOVF = isotropic volume fraction. *5 = 5‐percentile value of * signal. *95 = 95‐percentile value of * signal. Values correspond to the best cut‐off according to the Youden index. AUC = area under the receiver operating characteristic curve. NPV = negative predictive value, PPV = positive predictive value.

### Diagnostic Performances About the Grading Gliomas of Diffusion and Amide Proton Transfer Metrics

For glioma grading, except FA value from DKI and DTI models, almost every parameter derived from APT, DTI, DKI, NODDI, and MAP had significant differences between two of the three groups, that is, between glioma II and glioma III, between glioma II and glioma IV, or between glioma III and glioma IV (Appendix 3 of the Supplementary Material). Of these parameters, only the APT_5_ was significantly different at every comparison: between glioma II and glioma III (1.35 ± 0.75 vs. 2.09 ± 0.93), between glioma II and glioma IV (1.35 ± 0.75 vs. 2.71 ± 0.81), and between glioma III and glioma IV (2.09 ± 0.93 vs. 2.71 ± 0.81).

Table 4 displays the related AUC, sensitivity, specificity, positive predictive, and negative predictive values of the combined APT, DKI, DTI, MAP and NODDI models in comparisons of two of the three grading groups, and their corresponding ROCs are shown in Fig. 5b–d. There is no significant difference in differentiating different grading glioma for these models (*P* = 0.053–1), as indicated in Table 5.

**TABLE 4:** Performance of APT, DKI, DTI, MAP and NODDI Models in the Comparison of Grade II, Grade III, and Grade IV

Models	Comparison	AUC	Accuracy (%)	Sensitivity (%)	Specificity (%)	PPV (%)	NPV (%)
APT	II vs. III	0.729	73.53	85.00	57.14	73.91	72.73
	II vs. IV	0.924	85.37	81.48	92.86	95.65	72.22
	III vs. IV	0.705	77.08	89.29	60.00	75.76	80.00
DKI	II vs. III	0.761	73.53	60.00	92.86	92.31	61.90
	II vs. IV	0.911	90.24	92.59	85.71	92.59	85.71
DTI	II vs. III	0.829	79.41	70.00	92.86	93.33	68.42
	II vs. IV	0.924	97.80	85.19	92.86	95.83	76.47
	III vs. IV	0.695	70.83	60.71	85.00	85.00	60.71
MAP	II vs. III	0.714	73.53	80.00	64.29	76.19	69.23
	II vs. IV	0.964	92.68	92.59	92.86	96.15	86.67
NODDI	II vs. III	0.875	85.29	90.00	78.57	85.71	84.62
	II vs. IV	0.895	97.80	96.30	71.43	86.67	90.91
	III vs. IV	0.655	66.67	57.14	80.00	80.00	57.14

APT = amide proton transfer. DKI = diffusion kurtosis imaging. DTI = diffusion tensor imaging. MAP = mean apparent propagator. NODDI = neurite orientation dispersion and density imaging. vs. = versus. AUC = area under the receiver operating characteristic curve. PPV = positive predictive value. NPV = negative predictive value.

**TABLE 5:** Delong Test of Combined All Considered Parameters Derived From APT, DKI, DTI, MAP and NODDI in the Gliomas IDH Decoding and Grading

Comparison/*P* value Delong's Test^a^	IDH‐Mutant vs. IDH‐Wildtype	Grade II vs. Grade III	Grade II vs. Grade IV	Grade III vs. Grade IV
APT vs. DKI	0.045*	0.777	0.802	‐
APT vs. DTI	‐	0.271	1.000	0.935
APT vs. MAP	‐	0.905	0.379	‐
APT vs. NODDI	0.018*	0.110	0.566	0.661
DKI vs. DTI	‐	0.278	0.658	‐
DKI vs. MAP	‐	0.381	0.393	‐
DKI vs. NODDI	0.983	0.100	0.728	‐
DTI vs. MAP	‐	0.121	0.503	‐
DTI vs. NODDI	‐	0.393	0.358	0.751
MAP vs. NODDI	‐	0.053	0.231	‐

IDH = isocitrate dehydrogenase. APT = amide proton transfer. DKI = diffusion kurtosis imaging. DTI = diffusion tensor imaging. MAP = mean apparent propagator. NODDI = neurite orientation dispersion and density imaging. vs. = versus.

^a^ Test for comparison the difference of area under the receiver operating characteristic curve.

* *P* < 0.05. – Delong Test not applicable.

## Discussion

This study investigated the ability of DTI, DKI, NODDI, MAP, and APT imaging for gliomas grading and IDH status decoding using histopathological grades and genetic testing as standards. The parameters derived from APTw (APT_mean_, APT_5_, APT_95_), DKI (AD_95_, RD_95_, MD_95_), and NODDI (ISOVF_95_) were statistically different between the IDH‐wildtype and IDH‐mutant, and the APT_5_ value was the single index for simultaneously distinguishing glioma with three different grading. In addition, the results indicated that APT had higher diagnostic accuracy than DTI, DKI, and NODDI, suggesting that APT may be a valuable imaging biomarker in glioma grading and IDH status decoding.

Regarding the APT signals for IDH genotype decoding, we found that mean APTw value was significantly higher in IDH wildtype. According to the CEST theory, APT imaging can provide contrast correlated with metabolite concentrations and tumor cellularity based on the cellular mobile proteins and peptides.^23^, ^24^ The present study with a larger cohort was in line with previous studies in the diagnostic performance.^9^, ^25^, ^26^ In addition, in most studies, the APT MRI used a two‐dimensional single‐slice TSE approach, which could not provide coverage of the whole tumor. Conventional TSE acquisitions are limited by acquisition speed, making applications in clinical examinations difficult.^27^ This study used a whole‐brain isotropic CEST sequence by the three‐dimensional TSE readout sequence, also known as SPACE.^28^ This CEST‐SPACE technique can rapidly generate whole‐brain CEST source images with negligible susceptibility artifacts, and greatly improve the image quality of APTw.^10^, ^29^ These may impact clinical applications using APT because less time is required to obtain whole brain CEST source images than conventional TSE acquisitions.^10^

The APT_mean_ and APT_95_ values did not significantly distinguish glioma grading for APTw imaging in multiple comparisons, while the APT_5_ did. APTw imaging can generate contrast which, to a large extent, depends on the concentration of endogenous cellular proteins in tissue and the exchange properties of their amide protons with water protons (pH dependent), while other parameters, such as tissue water content, T1 value of water, and saturation efficiency, affect the contrast.^9^ Due to these factors, the gliomas with microscopic necrosis and cystic cavities have shown unstable APT signal intensity due to the very long T1 relaxation and inefficient saturation effect.^30^ Consistent with previous studies, the APTw image in our study showed that the average signal intensity of the cystic cavities, similar to tumor cores, was significantly stronger than those of the necrosis, immediate edema, and peripheral edema.^31^ Gliomas are prone to necrosis and cystic cavities.^32^ Therefore, even though the core tumor ROI was carefully delineated, it is challenging to avoid the ROI containing necrosis and cystic cavities entirely due to the low resolution of APT imaging, which leads to the overestimation of the APT_mean_ and APT_95_. Thus, APT_5_ became a more sensitive indicator, almost unaffected by tumor necrosis and cystic cavities.^22^, ^33^

Diffusion‐weighted imaging has been demonstrated to be beneficial in grading gliomas, which was consistent with a number of earlier investigations.^34^, ^35^ However, most studies only utilized one or two advanced diffusion models and categorized gliomas into high and low grades. Moreover, the results were less consistent in studies that performed multiple comparisons of grades II–IV.^26^, ^31^, ^36^ This study processed four advanced diffusion models through one approximately 5‐minute diffusion acquisition and compared the diagnostic accuracy of different advanced diffusion models for glioma grading. The results of this combined comparison are more informative and representative than those of individual investigations. Consistent with the results from most previous studies, no single DWI parameter can distinguish grades II–IV simultaneously. In addition, our results showed that there was no significant difference in the diagnostic performance between the four models combined with various parameters, which suggests that the advanced diffusion modeling may have limited benefit for grading across multiple tumor grades.^37^, ^38^, ^39^

Recent advancements in neuro‐oncology have changed the focus away from histopathologic grading and toward molecular characteristics, which have been incorporated into the WHO classification.^40^ The most crucial information neuroradiologists asked is not only about grading but also about molecular characteristics, especially in nonenhancing lower‐grade gliomas.^12^ Imaging must follow this paradigm shift and expand its scope to identify molecular status. Advanced MR imaging techniques may identify patients who benefit from early detection and intensive treatment. A key novelty of this study is that we used a novel 3D CEST sequence and four advanced diffusion models to compare the diagnosis performance and found that the APT_5_ value can identify tumor grading and IDH status. We are propelled to find out which imaging model or metric can offer more clinical benefits in future studies.

### Limitations

First, because our study was performed in a single center with a small number of patients, in the group of WHO grade IV glioma, only four patients had IDH‐mutant. It would be more persuadable to proceed with a subsequent study with multiple‐centers and a larger number of patients. Second, the ROIs were manually placed in solid parts of tumors by visual inspection and then automatically transferred to the T1‐weighted images space. However, some tumors located in the skull base or tissue–air/tissue–bone interface might affect the registration quality because of much more distortion in diffusion images, even though we had done registration between anatomic and diffusion images during DWI postprocessing and carefully checked during delineating ROIs. The position deviation between diffusion images and anatomical images is still difficult to completely avoid. Third, the resolution of APT and diffusion images is much lower than T1‐weighted MPRAGE, which leads to ROI containing other elements with partial volume effect. Finally, both APT and advanced diffusion models are complementary to conventional MR sequences. It would be of greater value to combine them with conventional sequences in diagnostic efficacy evaluation.

## Conclusion

Both APT and diffusion models are useful in glioma grading and IDH genotype status decoding. APT has higher diagnostic accuracy than DTI, DKI, MAP, and NODDI in differentiating glioma IDH genotype. APT_5_ is a parameter that can identify both tumor grading and IDH status decoding across all parameters generated from APT, DTI, DKI, MAP, and NODDI, making it a promising biomarker for tumor classification.

## Supporting information


**Appendix S1** Supporting information
