# Supplementary material for: Diagnostic performance of gliomas grading and IDH status decoding A comparison between 3D amide proton transfer APT and four diffusion‐weighted MRI models
Source: J Magn Reson Imaging. 2022 Apr 30;56(6):1834–44. doi: 10.1002/jmri.28211 (PMC9790544; doi:10.1002/jmri.28211)
Supplement: Supplementary file 1 — Appendix S1 Supporting information [file JMRI-56-1834-s001.docx]

**Appendix 1.** The parameters derived from APT, DTI, DKI, MAP and NODDI (mean ± standard deviation) in the different IDH status

| **Parameters** | | **IDH-wildtype** | **IDH-mutant** | **P value** | **Parameters** | | **IDH-wildtype** | **IDH-mutant** | **P value** |
| --- | --- | --- | --- | --- | --- | --- | --- | --- | --- |
| APT | Mean | 3.38±1.02 | 1.93±0.77 | <0.0001*** | MSD | Mean | 29.66±7.86 | 27.29±7.27 | 0.276 |
|  | P5 | 2.45±0.94 | 1.6±0.84 | 0.002* |  | P5 | 21.81±6.23 | 21.49±6.67 | 0.855 |
|  | P95 | 4.3±1.19 | 2.91±1.22 | <0.001** |  | P95 | 38.46±9.3 | 33.84±8.54 | 0.075 |
| DKI_AD  (10^-3^ mm^2^/s) | Mean | 1.67±0.43 | 1.51±0.44 | 0.179 | NG | Mean | 0.18±0.05 | 0.18±0.06 | 0.885 |
|  | P5 | 1.21±0.37 | 1.18±0.41 | 0.779 |  | P5 | 0.14±0.05 | 0.14±0.05 | 0.823 |
|  | P95 | 2.2±0.5 | 1.88±0.49 | 0.023* |  | P95 | 0.22±0.06 | 0.22±0.07 | 0.893 |
| DKI_AK | Mean | 0.68±0.17 | 0.7±0.2 | 0.699 | NGax | Mean | 0.15±0.04 | 0.15±0.05 | 0.753 |
|  | P5 | 0.54±0.12 | 0.57±0.16 | 0.408 |  | P5 | 0.11±0.04 | 0.12±0.04 | 0.610 |
|  | P95 | 0.85±0.26 | 0.85±0.27 | 0.965 |  | P95 | 0.19±0.05 | 0.19±0.06 | 0.980 |
| DKI_FA | Mean | 0.15±0.07 | 0.14±0.05 | 0.531 | NGrad | Mean | 0.1±0.03 | 0.1±0.04 | 0.956 |
|  | P5 | 0.08±0.04 | 0.08±0.03 | 0.961 |  | P5 | 0.08±0.02 | 0.08±0.03 | 0.702 |
|  | P95 | 0.24±0.1 | 0.22±0.07 | 0.497 |  | P95 | 0.13±0.04 | 0.12±0.04 | 0.571 |
| DKI_MD  (10^-3^ mm^2^/s) | Mean | 1.46±0.43 | 1.32±0.36 | 0.225 | QIV | Mean | 127.06±100.05 | 112.11±84 | 0.579 |
|  | P5 | 1.06±0.34 | 1.03±0.33 | 0.698 |  | P5 | 68.48±55.23 | 65.18±53.51 | 0.831 |
|  | P95 | 1.93±0.48 | 1.64±0.41 | 0.031* |  | P95 | 208.59±166.13 | 176.11±130.94 | 0.462 |
| DKI_MK | Mean | 0.7±0.17 | 0.72±0.19 | 0.709 | RTAP | Mean | 3.03±1.28 | 3.16±1.16 | 0.725 |
|  | P5 | 0.58±0.13 | 0.61±0.16 | 0.397 |  | P5 | 2.18±0.87 | 2.39±0.87 | 0.395 |
|  | P95 | 0.85±0.22 | 0.85±0.24 | 0.991 |  | P95 | 4.09±1.91 | 4.18±1.57 | 0.862 |
| DKI_RD  (10^-3^ mm^2^/s) | Mean | 1.35±0.43 | 1.21±0.34 | 0.232 | RTOP | Mean | 1.8±1.12 | 1.91±1.14 | 0.748 |
|  | P5 | 0.97±0.34 | 0.93±0.28 | 0.675 |  | P5 | 1.1±0.66 | 1.26±0.8 | 0.418 |
|  | P95 | 1.8±0.5 | 1.52±0.39 | 0.039* |  | P95 | 2.78±1.98 | 2.83±1.68 | 0.925 |
| DKI_RK | Mean | 0.72±0.17 | 0.75±0.19 | 0.597 | RTPP | Mean | 4.53±0.76 | 4.68±0.86 | 0.516 |
|  | P5 | 0.58±0.13 | 0.62±0.16 | 0.326 |  | P5 | 3.87±0.54 | 4.12±0.67 | 0.123 |
|  | P95 | 0.88±0.22 | 0.89±0.21 | 0.797 |  | P95 | 5.34±1.19 | 5.35±1.11 | 0.975 |
| DTI_AD  (10^-3^ mm^2^/s) | Mean | 0.97±0.27 | 0.94±0.32 | 0.717 | ICVF | Mean | 0.38±0.18 | 0.36±0.19 | 0.770 |
|  | P5 | 0.7±0.21 | 0.75±0.29 | 0.493 |  | P5 | 0.26±0.16 | 0.26±0.16 | 0.976 |
|  | P95 | 1.26±0.34 | 1.19±0.38 | 0.470 |  | P95 | 0.53±0.23 | 0.49±0.24 | 0.498 |
| DTI_FA | Mean | 0.15±0.07 | 0.15±0.06 | 0.998 | ISOVF | Mean | 0.29±0.18 | 0.2±0.12 | 0.065 |
|  | P5 | 0.08±0.04 | 0.09±0.04 | 0.563 |  | P5 | 0.12±0.13 | 0.08±0.08 | 0.337 |
|  | P95 | 0.23±0.1 | 0.23±0.08 | 0.995 |  | P95 | 0.49±0.23 | 0.34±0.16 | 0.017* |
| DTI_MD  10^-3^ mm^2^/s) | Mean | 0.85±0.26 | 0.82±0.27 | 0.637 | ODI | Mean | 0.51±0.16 | 0.47±0.19 | 0.478 |
|  | P5 | 0.63±0.19 | 0.64±0.23 | 0.762 |  | P5 | 0.32±0.16 | 0.32±0.18 | 0.867 |
|  | P95 | 1.11±0.33 | 1.03±0.34 | 0.406 |  | P95 | 0.7±0.16 | 0.63±0.19 | 0.163 |
| DTI_RD  (10^-3^ mm^2^/s) | Mean | 0.79±0.26 | 0.75±0.24 | 0.539 |  |  |  |  |  |
|  | P5 | 0.58±0.18 | 0.58±0.19 | 0.994 |  |  |  |  |  |
|  | P95 | 1.03±0.33 | 0.94±0.31 | 0.313 |  |  |  |  |  |

Abbreviations: IDH= isocitrate dehydrogenase. APT= amide proton transfer. DTI = diﬀusion tensor imaging. DKI = diffusion kurtosis imaging. NODDI = neurite orientation dispersion and density imaging. MAP = mean apparent propagator. AD = axial diffusivity. AK= axial kurtosis. FA= fractional anisotropy. MD = mean diffusivity. MK= mean kurtosis. RD = radial diffusivity. RK = radial kurtosis. MSD= mean squared displacement. NG= mean non-Gaussianity. NGAx = axial non-Gaussianity. NGRad = radial non-Gaussianity. QIV = q-space inverse variance. RTAP = return to the axis probability. RTOP= return-to-origin probability. RTPP= return-to-plane probability. ICVF= intracellular volume fraction. ISOVF= isotropic volume fraction. ODI = orientation dispersion index. *_5_ = 5-percentile value of * signal. *_95_ = 95-percentile value of * signal. vs.=versus. *P<0.05. **P<0.001. ***P<0.0001.

|  |  |  |  |  |  |  |  |  |  |  |  |  |  |  |  |  |  |
| --- | --- | --- | --- | --- | --- | --- | --- | --- | --- | --- | --- | --- | --- | --- | --- | --- | --- |
|  |  |  |  |  |  |  |  |  |  |  |  |  |  |  |  |  |  |
|  |  |  |  |  |  |  |  |  |  |  |  |  |  |  |  |  |  |

**Appendix 2.** Delong Test of APT, DKI and NODDI for All Comparisons and All Considered Parameters in the Prediction of IDH genotype

| **Model1ROC** | **Model2.ROC** | **Pvalue.Delong.test** | **Model1ROC** | **Model2.ROC** | **Pvalue.Delong.test** |
| --- | --- | --- | --- | --- | --- |
| APT_mean_ | APT_5_ | 0.023* | APT_95_ | NODDI_ISOVF_95_ | 0.268 |
| APT_mean_ | APT_95_ | 0.145 | APT_95_ | APT_all_ | 0.137 |
| APT_mean_ | DKI_AD_95_ | 0.062 | APT_95_ | DKI_all_ | 0.318 |
| APT_mean_ | DKI_MD_95_ | 0.044* | DKI_AD_95_ | DKI_MD_95_ | 0.664 |
| APT_mean_ | DKI_RD_95_ | 0.040* | DKI_AD_95_ | DKI_RD_95_ | 0.723 |
| APT_mean_ | NODDI_ISOVF_95_ | 0.020* | DKI_AD_95_ | NODDI_ISOVF_95_ | 0.898 |
| APT_mean_ | APT_all_ | 0.472 | DKI_AD_95_ | APT_all_ | 0.056 |
| APT_mean_ | DKI_all_ | 0.054 | DKI_AD_95_ | DKI_all_ | 0.408 |
| APT_5_ | APT_95_ | 0.222 | DKI_MD_95_ | DKI_RD_95_ | 0.899 |
| APT_5_ | DKI_AD_95_ | 0.678 | DKI_MD_95_ | NODDI_ISOVF_95_ | 0.983 |
| APT_5_ | DKI_MD_95_ | 0.617 | DKI_MD_95_ | APT_all_ | 0.043* |
| APT_5_ | DKI_RD_95_ | 0.592 | DKI_MD_95_ | DKI_all_ | 1.000 |
| APT_5_ | NODDI_ISOVF_95_ | 0.595 | DKI_RD_95_ | NODDI_ISOVF_95_ | 0.950 |
| APT_5_ | APT_all_ | 0.024* | DKI_RD_95_ | APT_all_ | 0.038* |
| APT_5_ | DKI_all_ | 0.633 | DKI_RD_95_ | DKI_all_ | 0.930 |
| APT_95_ | DKI_AD_95_ | 0.371 | NODDI_ISOVF_95_ | APT_all_ | 0.018* |
| APT_95_ | DKI_MD_95_ | 0.327 | NODDI_ISOVF_95_ | DKI_all_ | 0.983 |
| APT_95_ | DKI_RD_95_ | 0.302 | APT_all_ | DKI_all_ | 0.045* |

Abbreviations: APT= amide proton transfer. IDH= isocitrate dehydrogenase. DKI = diffusion kurtosis imaging. NODDI = neurite orientation dispersion and density imaging. RD = radial diffusivity. AD = axial diffusivity. MD = mean diffusivity. ISOVF= isotropic volume fraction. *_5_ = 5-percentile value of * signal. *_95_ = 95-percentile value of * signal. * P < 0.05.

**Appendix 3.** P values of all Comparisons which based on all APT, DTI, DKI, MAP and NODDI metrics in the gliomas grading

| **Parameters** | | **Grade II** | **Grade III** | **Grade IV** | **Grade II vs Grade III**  **(P value)** | | **Grade II vs Grade IV**  **(P value)** | **Grade III vs Grade IV**  **(P value)** |
| --- | --- | --- | --- | --- | --- | --- | --- | --- |
| APT | Mean | 2.15±0.8 | 2.81±1.25 | 3.47±0.99 | | 0.076 | <0.001** | 0.034* |
|  | P5 | 1.35±0.75 | 2.09±0.93 | 2.71±0.81 | | 0.014* | <0.0001*** | 0.014* |
|  | P95 | 2.85±1.13 | 3.84±1.55 | 4.45±0.94 | | 0.021* | <0.001** | 0.089 |
| DKI_AD | Mean | 1.92±0.41 | 1.58±0.44 | 1.51±0.39 | | 0.019* | 0.003* | 0.574 |
|  | P5 | 1.44±0.33 | 1.24±0.39 | 1.07±0.34 | | 0.098 | 0.002* | 0.107 |
|  | P95 | 2.33±0.51 | 2.05±0.53 | 2.04±0.48 | | 0.122 | 0.081 | 0.906 |
| DKI_AK | Mean | 0.57±0.15 | 0.7±0.18 | 0.73±0.17 | | 0.037* | 0.005* | 0.464 |
|  | P5 | 0.48±0.11 | 0.58±0.14 | 0.57±0.13 | | 0.032* | 0.030* | 0.898 |
|  | P95 | 0.69±0.21 | 0.83±0.29 | 0.94±0.23 | | 0.100 | 0.003* | 0.129 |
| DKI_FA | Mean | 0.14±0.04 | 0.14±0.07 | 0.16±0.07 | | 0.661 | 0.442 | 0.250 |
|  | P5 | 0.08±0.03 | 0.08±0.04 | 0.09±0.04 | | 0.742 | 0.737 | 0.444 |
|  | P95 | 0.21±0.06 | 0.23±0.11 | 0.24±0.1 | | 0.502 | 0.337 | 0.783 |
| DKI_MD | Mean | 1.66±0.44 | 1.39±0.42 | 1.31±0.36 | | 0.058 | 0.011* | 0.058 |
|  | P5 | 1.27±0.3 | 1.09±0.31 | 0.92±0.31 | | 0.097 | 0.001* | 0.068 |
|  | P95 | 2.01±0.49 | 1.79±0.49 | 1.8±0.46 | | 0.190 | 0.190 | 0.923 |
| DKI_MK | Mean | 0.6±0.15 | 0.72±0.18 | 0.76±0.16 | | 0.041* | 0.005* | 0.422 |
|  | P5 | 0.51±0.12 | 0.61±0.15 | 0.61±0.13 | | 0.042* | 0.029* | 0.978 |
|  | P95 | 0.71±0.19 | 0.84±0.24 | 0.93±0.2 | | 0.097 | 0.002* | 0.129 |
| DKI_RD | Mean | 1.54±0.45 | 1.27±0.42 | 1.22±0.35 | | 0.062 | 0.017* | 0.628 |
|  | P5 | 1.15±0.28 | 0.98±0.31 | 0.84±0.3 | | 0.117 | 0.002* | 0.096 |
|  | P95 | 1.88±0.52 | 1.62±0.5 | 1.7±0.46 | | 0.132 | 0.262* | 0.584 |
| DKI_RK | Mean | 0.62±0.16 | 0.73±0.18 | 0.78±0.16 | | 0.069 | 0.007* | 0.353 |
|  | P5 | 0.52±0.13 | 0.61±0.15 | 0.62±0.13 | | 0.067 | 0.032* | 0.816 |
|  | P95 | 0.76±0.18 | 0.87±0.22 | 0.95±0.2 | | 0.141 | 0.005* | 0.150 |
| DTI_AD | Mean | 1.19±0.27 | 0.95±0.26 | 0.87±0.25 | | 0.010* | <0.001** | 0.283 |
|  | P5 | 0.87±0.24 | 0.75±0.21 | 0.62±0.2 | | 0.100 | 0.001* | 0.045* |
|  | P95 | 1.47±0.35 | 1.21±0.36 | 1.14±0.3 | | 0.029* | 0.003* | 0.460 |
| DTI_FA | Mean | 0.14±0.05 | 0.14±0.06 | 0.15±0.07 | | 0.909 | 0.512 | 0.386 |
|  | P5 | 0.09±0.04 | 0.07±0.03 | 0.09±0.05 | | 0.356 | 0.917 | 0.226 |
|  | P95 | 0.22±0.07 | 0.22±0.1 | 0.23±0.11 | | 0.987 | 0.811 | 0.774 |
| DTI_MD | Mean | 1.02±0.28 | 0.83±0.24 | 0.76±0.22 | | 0.032* | 0.002* | 0.285 |
|  | P5 | 0.75±0.19 | 0.66±0.18 | 0.55±0.19 | | 0.183 | 0.002* | 0.054 |
|  | P95 | 1.25±0.38 | 1.08±0.34 | 1±0.28 | | 0.120 | 0.019* | 0.420 |
| DTI_RD | Mean | 0.94±0.27 | 0.78±0.25 | 0.7±0.22 | | 0.053 | 0.003* | 0.270 |
|  | P5 | 0.67±0.16 | 0.6±0.17 | 0.51±0.18 | | 0.226 | 0.006* | 0.094 |
|  | P95 | 1.16±0.38 | 1±0.34 | 0.92±0.27 | | 0.158 | 0.025* | 0.389 |
| MSD | Mean | 33.74±7.96 | 28.39±7.57 | 27±6.88 | | 0.041* | 0.007* | 0.521 |
|  | P5 | 25.31±5.37 | 22.26±6.27 | 19.53±6.04 | | 0.148 | 0.004* | 0.124 |
|  | P95 | 40.74±10.05 | 36.5±9.69 | 35.74±8.38 | | 0.191 | 0.102 | 0.779 |
| NG | Mean | 0.15±0.05 | 0.18±0.05 | 0.19±0.05 | | 0.073 | 0.009* | 0.397 |
|  | P5 | 0.12±0.04 | 0.15±0.05 | 0.15±0.05 | | 0.106 | 0.049* | 0.775 |
|  | P95 | 0.19±0.06 | 0.22±0.06 | 0.24±0.06 | | 0.183 | 0.006* | 0.116 |
| NGax | Mean | 0.12±0.04 | 0.15±0.04 | 0.16±0.04 | | 0.071 | 0.008* | 0.376 |
|  | P5 | 0.1±0.04 | 0.12±0.04 | 0.12±0.04 | | 0.128 | 0.050* | 0.694 |
|  | P95 | 0.16±0.05 | 0.18±0.05 | 0.2±0.05 | | 0.168 | 0.009* | 0.176 |
| NGrad | Mean | 0.08±0.03 | 0.1±0.03 | 0.11±0.03 | | 0.103 | 0.011* | 0.331 |
|  | P5 | 0.07±0.02 | 0.08±0.02 | 0.08±0.03 | | 0.164 | 0.059 | 0.636 |
|  | P95 | 0.1±0.04 | 0.12±0.04 | 0.14±0.04 | | 0.167 | 0.004* | 0.106 |
| QIV | Mean | 189.63±120.65 | 115.64±89.29 | 94.31±68.38 | | 0.020* | 0.002* | 0.416 |
|  | P5 | 104.84±74.03 | 67.5±40.87 | 48.88±42.07 | | 0.038* | 0.001* | 0.213 |
|  | P95 | 292.93±209.71 | 189.48±158.22 | 159.19±100.85 | | 0.052 | 0.008* | 0.492 |
| RTAP | Mean | 2.39±0.86 | 3.16±1.3 | 3.35±1.25 | | 0.073 | 0.018* | 0.587 |
|  | P5 | 1.83±0.6 | 2.4±0.88 | 2.34±0.93 | | 0.057 | 0.072 | 0.796 |
|  | P95 | 3.19±1.15 | 4.12±2.03 | 4.58±1.77 | | 0.131 | 0.018* | 0.367 |
| RTOP | Mean | 1.25±0.7 | 1.93±1.29 | 2.06±1.09 | | 0.082 | 0.028* | 0.685 |
|  | P5 | 0.83±0.42 | 1.29±0.77 | 1.21±0.73 | | 0.062 | 0.099 | 0.698 |
|  | P95 | 1.98±1.32 | 2.81±2.33 | 3.2±1.69 | | 0.207 | 0.049* | 0.469 |
| RTPP | Mean | 4.09±0.66 | 4.63±0.82 | 4.78±0.74 | | 0.044* | 0.007* | 0.512 |
|  | P5 | 3.66±0.48 | 4.06±0.65 | 3.99±0.56 | | 0.049* | 0.081 | 0.681 |
|  | P95 | 4.65±0.86 | 5.27±1.28 | 5.74±1.05 | | 0.112 | 0.003* | 0.142 |
| ICVF | Mean | 0.28±0.17 | 0.36±0.17 | 0.43±0.18 | | 0.206 | 0.010* | 0.149 |
|  | P5 | 0.19±0.13 | 0.25±0.14 | 0.3±0.17 | | 0.215 | 0.027* | 0.303 |
|  | P95 | 0.43±0.25 | 0.48±0.22 | 0.6±0.22 | | 0.527 | 0.022* | 0.066 |
| ISOVF | Mean | 0.34±0.21 | 0.2±0.16 | 0.27±0.14 | | 0.023* | 0.201 | 0.190 |
|  | P5 | 0.16±0.16 | 0.09±0.11 | 0.09±0.08 | | 0.074 | 0.049* | 0.933 |
|  | P95 | 0.51±0.25 | 0.35±0.2 | 0.48±0.21 | | 0.036* | 0.651 | 0.045* |
| ODI | Mean | 0.39±0.18 | 0.53±0.16 | 0.52±0.16 | | 0.019* | 0.020* | 0.835 |
|  | P5 | 0.24±0.15 | 0.35±0.17 | 0.35±0.16 | | 0.064 | 0.046* | 0.976 |
|  | P95 | 0.55±0.2 | 0.7±0.14 | 0.72±0.15 | | 0.009* | 0.002* | 0.705 |

Abbreviations: APT= amide proton transfer. DTI = diﬀusion tensor imaging. DKI = diffusion kurtosis imaging. NODDI = neurite orientation dispersion and density imaging. MAP = mean apparent propagator. AD = axial diffusivity. AK= axial kurtosis. FA= fractional anisotropy. MD = mean diffusivity. MK= mean kurtosis. RD = radial diffusivity. RK = radial kurtosis. MSD= mean squared displacement. NG= mean non-Gaussianity. NGAx = axial non-Gaussianity. NGRad = radial non-Gaussianity. QIV = q-space inverse variance. RTAP = return to the axis probability. RTOP= return-to-origin probability. RTPP= return-to-plane probability. ICVF= intracellular volume fraction. ISOVF= isotropic volume fraction. ODI = orientation dispersion index. *_5_ = 5-percentile value of * signal. *_95_ = 95-percentile value of * signal. vs=versus. *P<0.05. **P<0.001. ***P<0.0001.
